# Fluoroless left atrial access for radiofrequency and cryoballoon ablations using a novel radiofrequency transseptal wire

**DOI:** 10.1007/s10840-022-01157-5

**Published:** 2022-02-22

**Authors:** Hany Demo, Carla Aranda, Mansour Razminia

**Affiliations:** 1 Swedish Hospital, NorthShore University HealthSystem, Chicago, IL USA; 2 AMITA Health St. Joseph Hospital, 1975 Lin Lor Ln Ste. 155, Elgin, IL 60123 USA

**Keywords:** Catheter ablation, Arrhythmia, Fluoroless, Cryoablation, ICE, Electroanatomic mapping, Radiofrequency wire

## Abstract

**Purpose:**

Conventional catheter ablation for atrial fibrillation requires fluoroscopy, which has inherent risks of radiation exposure to patients and medical staff. Optimization of fluoroscopy parameters and use of three-dimensional electroanatomic mapping (EAM) and intracardiac echocardiography (ICE) have helped to reduce radiation exposure; however, despite growing evidence, there are still concerns about safety and added procedure time associated with fluoroless procedures, particularly in left-sided ablations, due to the potential risk of complications. Herein, we report our initial experience using a radiofrequency (RF) wire for completely fluoroless radiofrequency ablation (RFA) and cryoballoon ablation (CBA).

**Methods:**

A retrospective analysis was conducted on ablation procedures for various cardiac arrhythmias performed non-fluoroscopically at two centers using the VersaCross RF wire transseptal system under EAM and ICE guidance.

**Results:**

A total of 72 and 54 patients underwent RFA and CBA, respectively, successfully without any procedural complications. Transseptal access time for RFA was 14.5 ± 6.6 min from procedure start (including sheath and catheter placements ± right-sided ablation) or 2.8 ± 1.0 min from RF wire insertion into the femoral introducer. Transseptal access time for CBA was 19.2 ± 11.7 min from procedure start (including sheath and catheter placements ± right-sided ablation) or 3.5 ± 1.6 min from RF wire insertion into the femoral introducer. Average procedure time was 104.4 ± 38.0 min for RFA and 91.1 ± 22.1 min for CBA.

**Conclusions:**

A RF wire can be used to achieve completely fluoroless transseptal puncture safely and effectively while improving procedural efficiency in both RFA and CBA.

## Introduction

Catheter ablation of arrhythmias is conventionally performed using fluoroscopy as visual guidance for catheter placement. Given the common use of pre-procedural cardiac computed tomography scans for definition of cardiac and extracardiac anatomy, the long duration of catheter ablation procedures, and frequent need for repeat ablation procedures, the collective lifetime of radiation exposure can be significant for patients [1]. Increased exposure to radiation can lead to genetic defects [2], malignancies [2, 3], skin burns [4, 5], and cataracts [6]. Conventional catheter ablation procedures can expose patients to an average of 15 mSv of radiation, comparable to 750 chest X-rays [7]. The adoption of fluoroscopy with an ultralow framerate for ablation procedures has significantly reduced radiation exposure to 0.45 mSv [8]; however, medical staff still face significant lifetime cumulative effects of radiation. In addition to exposure to radiation hazards, medical staff also face orthopedic injuries due to the use of heavy protective lead apparel [9]. Up to 44% of interventional cardiologists and electrophysiologists that wear these heavy lead shields to protect themselves against radiation exposure experience spine, hip, knee, and ankle pain [9–11].

Several studies have demonstrated the use of 3-dimensional (3D) electroanatomic mapping (EAM) systems and intracardiac echocardiography (ICE) to decrease or completely eliminate fluoroscopy usage during catheter ablation [12–18]. Although the feasibility of transseptal puncture using EAM guidance has also been reported [19, 20], difficulty with visualizing the transseptal devices led to the use of fluoroscopy in 30 to 60% of the cases [17, 21]. Visualization of the transseptal needle during superior vena cava (SVC) cannulation, as well as while positioning on the interatrial septum, is crucial. Poor visualization during transseptal puncture can lead to serious complications. The visualization of a conventional needle on ICE or EAM is not performed as it requires the exposure of the sharp needle tip, presenting the risk of tissue injury and unintended puncture during dropdown and tenting. Recent studies have demonstrated that the use of a radiofrequency (RF) transseptal needle connecting with the 3D EAM system can safely allow for fluoroless catheter ablation procedures [13, 18, 22–27]. Despite growing evidence of the benefits of fluoroless catheter ablation, concern still remains regarding the safety and added procedure time associated with fluoroless procedures, mainly due to inability to confirm the location of the needle tip. A new RF transseptal wire has been shown to improve procedural efficiency by utilizing a J-tipped or pigtail RF wire to cannulate the SVC, perform transseptal puncture, and provide an exchange rail without any device exchanges [28, 29]. This novel RF wire can be easily visualized on 3D EAM system as well as ICE. Combining the efficiency of a RF wire with fluoroless visualization can improve adoption of a completely fluoroless laboratory without compromising safety and enhance efficiency. Here, we describe the first combined clinical experience using a RF wire-based transseptal system to improve procedural efficiency and facilitate zero-fluoroscopy radiofrequency ablation (RFA) and cryoballoon ablation (CBA).

## Methods

### Study design

A retrospective chart review was conducted on catheter ablation procedures for various left-sided cardiac arrhythmias performed consecutively at two different centers between July 2020 and June 2021. Zero-fluoroscopy RFA or CBA was performed by two operators (MR or HD, respectively), as previously described [30, 31], with standard informed consent obtained prior to procedures. Transseptal puncture for RFA was guided by EAM and ICE, while CBA used ICE guidance alone. Intraprocedural complications, procedure time, transseptal time, and fluoroscopy dose and time were recorded. Procedure start time was indicated by femoral venous access. Transseptal time and time to left atrial access with respect to procedure start included time for vascular access, sheaths, ICE catheter and coronary sinus (CS) catheter placement, as well as electrophysiology study, right atrial (RA) ablations, and/or echocardiographic measurements.

### Mapping and initial catheter placement

**Fig. 1:**
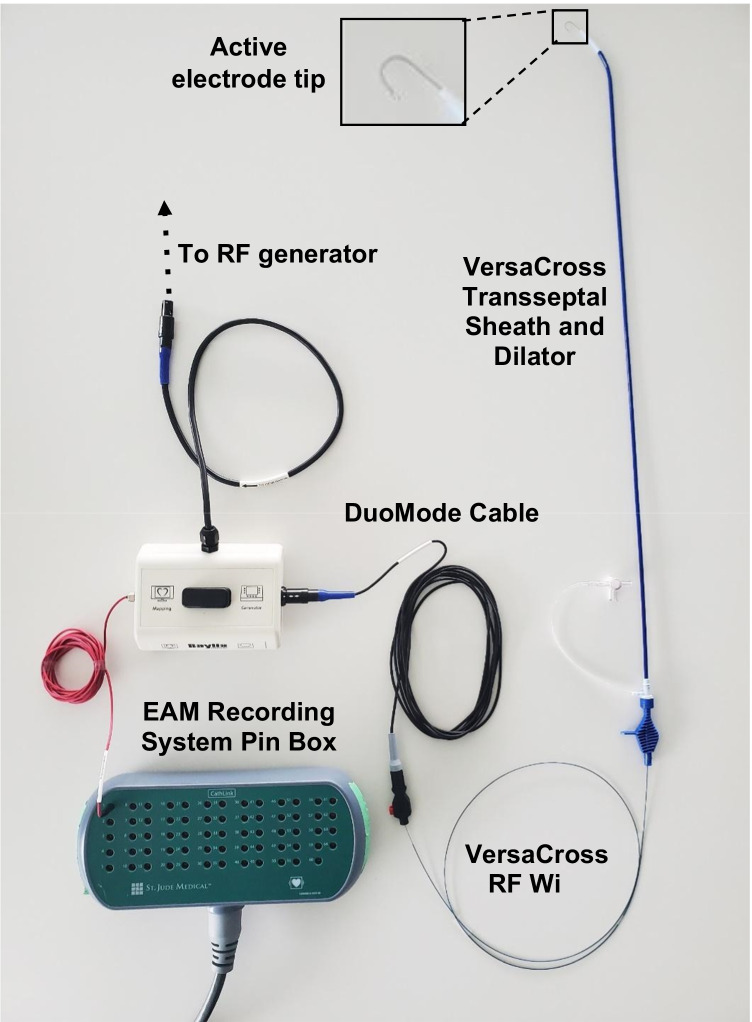
Set up for visualization of 0.035 RF wire (VersaCross RF Wire, Baylis Medical Company, Montreal QC, CA). A DuoMode extension cable (Baylis Medical) was used to connect the RF wire to the EnSite velocity cardiac mapping system pin box (Abbott, St. Paul, MN) to enable visualization during superior vena cava (SVC) cannulation, drop-down to the fossa ovalis, and transseptal puncture

Percutaneous femoral venous access was obtained using vascular ultrasound guidance. Two 8-Fr sheaths were placed in the right femoral vein; one 10-Fr long sheath and another 7-Fr sheath were placed in the left femoral vein. A 9-Fr ICE catheter (ViewFlex, Abbott, St. Paul, MN, USA) was inserted through the 10-Fr long sheath in the left femoral vein and advanced to the inferior vena cava (IVC) while maintaining an echo-free space at the tip of the transducer. The ICE catheter was then advanced toward the RA. A 6-Fr decapolar catheter (Inquiry Diagnostic Catheter, Abbott) was next inserted into the left femoral vein and into the IVC under EAM guidance (EnSite Precision, Abbott) to generate IVC, SVC, RA, and CS geometries. The 0.035-inch RF wire (VersaCross RF Wire, Baylis Medical, Montreal, QC, CA) was visualized on the EnSite system using “mapping mode” on the dedicated DuoMode extension cable (Baylis Medical) (Fig. 1).

### Transseptal access

**Fig. 2:**
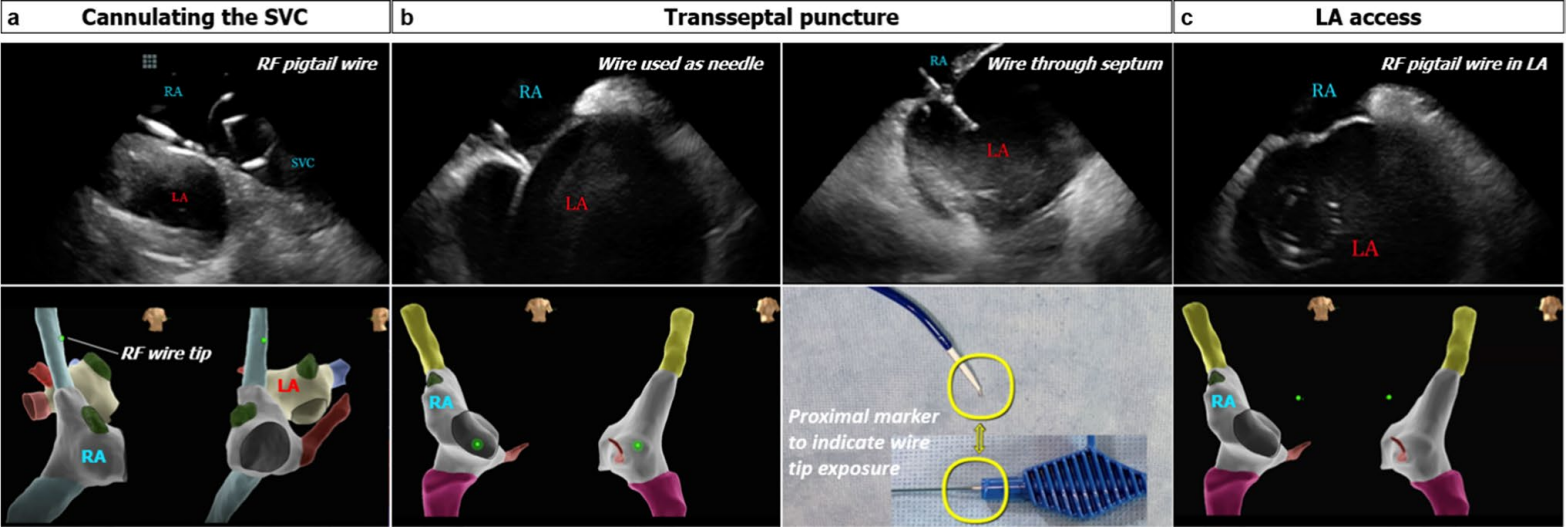
Visualization of RF wire with intracardiac echocardiography and electroanatomic mapping during (**a**) SVC cannulation, (**b**) transseptal puncture, and (**c**) LA access. Top panels: intracardiac echocardiography visualization of RF pigtail wire in SVC, RF wire tenting interatrial septum, RF wire through septum after RF energy application, and RF pigtail wire in LA. Bottom panels: electroanatomic visualization of RF wire tip in SVC, in the RA, and in the LA. SVC, superior vena cava; RA, right atrium; LA, left atrium

A weight-based heparin bolus was administered prior to transseptal puncture. Activated clotting time was monitored throughout the procedure, and intravenous heparin bolus was administered to target 350 to 450 s. A J-tip or pigtail RF wire (VersaCross RF Wire, Baylis Medical) was inserted into the right femoral vein and advanced until it was confirmed to be in the SVC under ICE (Fig. 2a). The short introducer was exchanged for a transseptal sheath and dilator (VersaCross Transseptal Sheath, Baylis Medical), which was advanced over the RF wire into the SVC under ICE visualization. The RF wire was withdrawn slightly to the third indicator such that the distal end of the RF wire aligned with the distal end of the dilator. The transseptal assembly and ICE catheter were then dropped down simultaneously into the RA and positioned on the interatrial septum. The RF wire tip was also tracked as a discrete point on the EAM system during dropdown (Fig. 2a). RF wire tip position and tenting of the fossa ovalis were confirmed on ICE (Fig. 2b) before performing transseptal puncture. Once position was deemed satisfactory, the DuoMode was switched to generator mode, and RF energy was delivered via the RF wire. Left atrial (LA) access was confirmed on ICE by visualizing the pigtail or J-tip RF wire across the septum and the appearance of microbubbles in the LA. The DuoMode was then switched back to mapping mode to allow tracking of the RF wire on the EAM. The wire visualization outside of the RA geometry confirmed LA access. A second transseptal puncture using the RF wire was performed in the same fashion in RFA cases. In both CBA and RFA, the ICE catheter was advanced into the LA to guide the ablation procedure.

### Radiofrequency catheter ablation

An esophageal temperature probe (CIRCA S-Cath, CIRCA Scientific, Englewood, CO, USA) was inserted into the esophagus, and the position was confirmed on ICE [25].

The RF wire and dilator were exchanged for an ablation catheter or circular mapping catheter. The geometry of the LA, pulmonary veins, and left atrial appendage was collected using EAM and ICE. RFA of each arrhythmia was performed using standard methods, whereby stable catheter position and contact during ablation was confirmed on ICE. EAM was used for catheter guidance and to track and monitor lesion formation.

### Cryoballoon ablation procedure

After transseptal puncture, the pigtail wire was maintained in the mid left atrium to maintain access. The sheath and dilator were exchanged for the FlexCath (FlexCath Advance, Medtronic, Minneapolis, MN, USA) over the wire. The 10.5-Fr 28-mm cryoballoon (Arctic Front Advance, Medtronic) and an 8-pole circular mapping catheter (Achieve, Medtronic) were placed through the FlexCath sheath into the LA for cryoballoon ablation, as per standard protocol. ICE guidance was used to confirm the alignment of sheath and balloon relative to the PV before cryoenergy application. Doppler imaging as well as hemodynamic pressure monitoring was used to confirm pulmonary vein occlusion.

## Results

### Patient characteristics

A total of 126 patients underwent catheter ablation for left-sided cardiac arrhythmias. RFA was performed on 72 patients, and CBA was performed on 54 patients. While most patients were treated for atrial fibrillation, catheter ablation was also performed for atrial flutter, mitral isthmus flutter, atrial tachycardia, atrioventricular nodal reentry tachycardia (AVNRT), premature ventricular contractions (PVC), and ventricular tachycardia (Table 1). The percentage of patients with more than one type of arrhythmia was 31%. History of prior transseptal puncture was 39% and 4% in the RFA and CBA group, respectively. Baseline characteristics were similar in both the RFA and CBA group (Table 1). The mean patient age was 67.6 ± 12.5, with a study population that was 51% male having a mean body mass index of 29.9 ± 7.2.

**Table 1:** Baseline patient characteristics

Characteristic	RF ablation	Cryoballoon ablation
	(*n* = 72)	(*n* = 54)
Age (avg., years)	69.2 ± 13.8	65.7 ± 10.3
Gender (male sex) (%)	51	50
BMI (avg.)	30.6 ± 6.6	29.1 ± 7.9
Cardiac arrhythmia (no. of patients)		
Atrial fibrillation	57	54
Atrial tachycardia	33	3
Atrial flutter	9	9
Other	6	0
AF classification (%)		
Paroxysmal	44	85
Persistent	23	13
Long-standing persistent	33	2
Hypertension (%)	79	91
Diabetes (%)	13	50
Coronary artery disease (%)	28	52
Ejection fraction (%)		
Normal (> 60%)	78	28
Borderline (50–60%)	5	57
Low (< 50%)	16	15
OAC use (%)	95	96
Implants/pacemakers (%)	14	33
History of prior transseptal puncture (%)	39	4

*BMI* body mass index, *OAC* oral anticoagulants. Other cardiac arrhythmias include AVNRT, PVC, ventricular tachycardia, mitral isthmus flutter

### Transseptal puncture

**Fig. 3:**
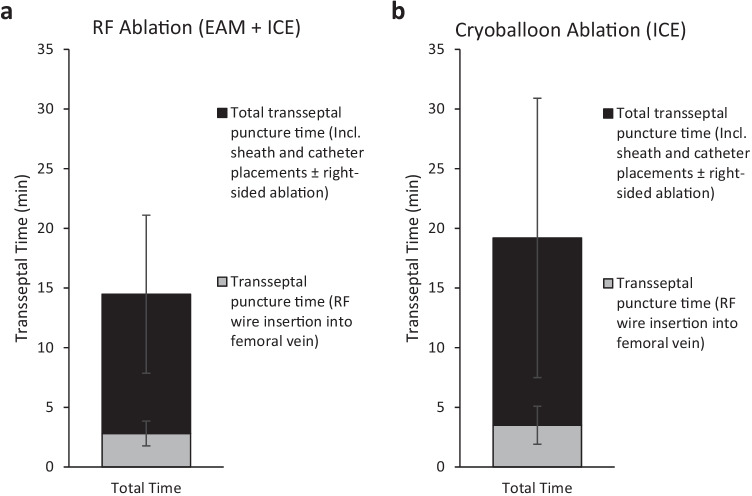
Transseptal puncture time. (**a**) Mean total transseptal puncture time from vascular access including sheath and catheter placements ± right-sided ablation and transseptal puncture time from RF wire insertion into femoral introducer (gray) during RF ablation using EAM and ICE for visualization (*n* = 72). (**b**) Mean total transseptal puncture time from vascular access including sheath and catheter placements ± right-sided ablation and transseptal puncture time from RF wire insertion into femoral introducer (gray) during cryoballoon ablation using ICE for visualization (*n* = 54)

Transseptal puncture was successful in 100% of cases without any fluoroscopy use and no intraoperative complications (Table 2). Double transseptal puncture was performed in 89% of RFA cases. Fluoroscopy was not used in any of the procedures. The average transseptal access time for all procedures was 16.1 ± 8.8 min from initial femoral access (procedure start time) or 3.0 ± 1.2 min from RF wire insertion into the femoral introducer. In RFA cases, transseptal access was achieved within 14.5 ± 6.6 min from initial vascular access (including sheath and catheter placements ± right-sided ablation) or 2.8 ± 1.0 min from insertion of the RF wire in the femoral introducer (Fig. 3). Transseptal access in CBA cases was achieved within 19.2 ± 11.7 min of vascular access (including sheath and catheter placements ± right-sided ablation) or 3.5 ± 1.6 min from RF wire insertion into femoral introducer.

**Table 2:** Procedural information

**Parameter**	Total (*n* = 126)	RF ablation (*n* = 72)	Cryoballoon ablation (*n* = 54)
*Transseptal puncture*			
Number of punctures (total)	190	136	54
Time to LA access (avg.; min)	16.1 ± 8.8	14.5 ± 6.6	19.2 ± 11.7
Fluoroscopy use			
Number of cases	0	0	0
Time (avg.; min)	0	0	0
Dose (mGy)	0	0	0
*PVI (AF ablations)*			
Overall procedure time (avg.; min)	100.8 ± 36.6 (*n* = 111)	104.4 ± 38.0 (*n* = 57)	91.1 ± 22.1 (*n* = 54)
Fluoroscopy use			
Number of cases	0	0	0
Time (avg.; min)	0	0	0
Dose (mGy)	0	0	0
Acute success (%)	100	100	100
Complications	0	0	0

### Ablation

Acute procedural success, defined as electrical isolation of individual pulmonary veins, was achieved in all cases without the use of fluoroscopy during RFA and CBA. Ablation of atrial fibrillation was performed in 79% of patients in the RFA group and 100% of the CBA. Ablation of primary or secondary atrial tachycardia, atypical atrial flutters, AVNRT, PVC, or ventricular tachycardia was performed in 67% of patients in the RFA group and 22% of patients in the CBA group. The average procedure time from initial transvenous access to final sheath removal for all PVI procedures was 100.8 ± 36.6 min. The average procedure time for PVI using RFA was 104.4 ± 38.0 min and 91.1 ± 22.1 min using CBA. No fluoroscopy was required during any cases. All procedures were concluded without any intraprocedural complications.

## Discussion

To our knowledge, this is the first case series to describe the safety and effectiveness of a completely fluoroless RFA and CBA technique using the novel RF wire for transseptal puncture. Our centers have previously established completely fluoroless ablation protocols using the RF needle. In the present study, in an attempt to evaluate the safety and efficiency of a new RF wire that can easily be visualized on 3D EAM system and ICE, we evaluated fluoroless transseptal puncture using the RF wire, 3D EAM, and/or ICE. Using the techniques described in this study, we achieved 100% transseptal success performing either a single or double transseptal puncture with zero-fluoroscopy use. This was achieved regardless of septal anatomy, prior history of transseptal catheterization, or the presence of cardiac implanted devices, and with no procedure-related complications. These results are similar to what has been observed using the RF needle [18]. With easy visualization of the RF wire using EAM and/or ICE, all procedures were conducted without any fluoroscopy from start to finish. The first transseptal puncture, including catheters placement, RA ablation, and/or echocardiographic measurements, took an average of 16 min from vascular access, which is an improvement relative to current standard methods using RF needle of up to 28 min [18], and significantly faster than conventional fluoroscopic procedures using a mechanical needle [32]. Transseptal puncture from RF wire insertion into the femoral introducer took an average of 3.0 min, with 2.8 min for RFA, and 3.5 min for CBA. These times are comparable to those seen with fluoroscopy-guided transseptal puncture using the RF wire [28], suggesting that fluoroless visualization techniques do not encumber transseptal puncture efficiency. All procedures were completed within an average of 1.7 h, which is less than or comparable to what has been previously reported [12–14, 33].

Although several labs have adopted fluoroless workflows, safety considerations and inadequate visualization have been limitations to more widespread adoption. This early multicenter clinical experience showed that the VersaCross RF wire improves procedural efficiency while enabling fluoroless visualization and maintaining procedural safety. The RF J-tip or pigtail wire is designed to be used as a starting guidewire which, combined with the RF electrode tip, can be uniquely visualized and tracked on EAM as soon as vascular access is obtained. This allows the wire to be tracked while cannulating the SVC, which is not otherwise possible using a standard mechanical needle or guidewire. The rounded atraumatic RF wire tip can be exposed from the dilator during dropdown and positioning on the fossa ovalis, similarly to the RF needle, allowing for a safe and controlled RF transseptal puncture even in difficult septal anatomies. In comparison, visualization of a conventional mechanical needle on ICE or EAM requires exposure of the sharp needle tip beyond the end of the dilator, presenting the risk of tissue injury and/or unintended puncture during dropdown and tenting. In the absence of an isolated electrode tip, a mechanical needle or guidewire cannot be visualized as a well-defined point on EAM and may lead to echocardiographic artifacts that hinder one or both transseptal punctures [34]. In fact, a recent study describing visualization of a sharp mechanical transseptal needle with EAM reported unsuccessful transseptal puncture in 10% of patients and a case of cardiac tamponade [19].

The VersaCross RF wire eliminated the initial guidewire exchange for a transseptal needle and, in cryoballoon ablations, the need for an exchange wire to introduce the FlexCath sheath. The RF wire also allowed direct rewiring into the SVC without requiring device exchanges. Avoiding extra device exchanges not only improves procedural efficiency but also reduces the risk of injury and air embolism [28, 35]. The use of a dedicated RF transseptal needle enables fluoroless technique and has significantly improved transseptal puncture success in a wide range of septal anatomies. The RF transseptal needle is considered a standard of practice in many electrophysiology labs owing to the improved safety and effectiveness profiles [32] and proven cost-effectiveness [36]. In our experience, we found that the new RF wire improved on the current standard by reducing device exchanges and improving procedural efficiency and has been implemented as the new standard of practice in our labs.

### Limitations

While this study demonstrated that fluoroless RFA and CBA can be safely and effectively performed using the new RF wire in limited early experience, this is not a comparative study, and time savings are estimated based on previously published data at different centers. All procedures were performed in two centers who have implemented a fully fluoroless protocol in their electrophysiology laboratories for several years. Although the safety features of the RF wire may make it easier and safer for new users to learn and practice completely fluoroless techniques, additional studies are needed using operators with different levels of experience to evaluate more widespread adoption of completely fluoroless procedures.

## Conclusions

Radiation risk to patients and medical staff during catheter ablation procedures is high, and efforts should be made to eliminate unnecessary exposure whenever possible. This study describes a technique for zero-fluoroscopy transseptal puncture for RF and cryoballoon catheter ablation of various cardiac arrhythmias. We demonstrated effective visualization of the RF wire with EAM and/or ICE, as well as reducing the number of device exchanges for LA access. This allowed for more efficient and faster catheter ablation procedure in a lead-free environment, as compared to conventional fluoroless techniques. This RF wire is now fully adopted in our centers for completely fluoroless ablations.
