# Correction to: Fluoroless left atrial access for radiofrequency and cryoballoon ablations using a novel radiofrequency transseptal wire

**DOI:** 10.1007/s10840-022-01185-1

**Published:** 2022-03-29

**Authors:** Hany Demo, Carla Aranda, Mansour Razminia

**Affiliations:** 1 Swedish Hospital, NorthShore University HealthSystem, Chicago, IL USA; 2 AMITA Health St. Joseph Hospital, 1975 Lin Lor Ln Ste. 155, Elgin, IL 60123 USA

**Correction to: Journal of Interventional Cardiac Electrophysiology**

**https://doi.org/10.1007/s10840-022-01157-5**

The article "Fluoroless left atrial access for radiofrequency and cryoballoon ablations using a novel radiofrequency transseptal wire", written by Hany Demo, Carla Aranda and Mansour Razminia, was originally published electronically on the publisher’s internet portal on 22 February 2022 without open access. With the author(s)’ decision to opt for Open Choice the copyright of the article changed on 28 February 2022 to © The Author(s) 2022 and the article is forthwith distributed under a Creative Commons Attribution 4.0 International License, which permits use, sharing, adaptation, distribution and reproduction in any medium or format, as long as you give appropriate credit to the original author(s) and the source, provide a link to the Creative Commons licence, and indicate if changes were made. The images or other third party material in this article are included in the article’s Creative Commons licence, unless indicated otherwise in a credit line to the material. If material is not included in the article’s Creative Commons licence and your intended use is not permitted by statutory regulation or exceeds the permitted use, you will need to obtain permission directly from the copyright holder.

The original article has been corrected.

